# Health Security: Definition Problems

**DOI:** 10.3390/ijerph191610009

**Published:** 2022-08-13

**Authors:** Anna Augustynowicz, Janusz Opolski, Michał Waszkiewicz

**Affiliations:** 1 Department of Economics of Health and Medical Law, Medical University of Warsaw, 02-091 Warsaw, Poland; 2 School of Public Health Centre of Postgraduate Medical Education of Warsaw, 01-813 Warsaw, Poland; 3 Faculty of Engineering and Management, University of Ecology and Management in Warsaw, 00-792 Warsaw, Poland

**Keywords:** health security, health system, safety, patient

## Abstract

The link between security and health is not a discovery. Despite the widespread recognition of the social importance of health security, there is no single common definition of this concept. The study’s objective was to search, analyse and evaluate possible original propositions to define the concept of “health security” in Polish professional literature published from 10 January 2017 to 31 March 2022. The research method was a scoping review performed in five stages according to H. Arksey and L. O’Malley. Ultimately, the study criteria were met by four studies. The proposed definition of health security in these papers failed to solve this problem and raised further questions and doubts. It is urgent to start a discourse on the meaning of the concept of “health security” with the broadest possible participation of representatives of various scientific disciplines, but taking into account the knowledge and practice of public health. It seems that it will be impossible to avoid the following questions: what is health security nowadays? What is health security as a personal issue? What are the necessary steps to achieve the consensus? Is it worthwhile to consider it on the international level?

## 1. Introduction

The link between security and health is not a discovery. However, in the past, this relationship was narrowly understood as the relationship between diseases and the military potential of states and/or as the impact of armed conflicts on human health and the functioning of health care [1].

The collapse of the Soviet Union and—as it seemed—the end of the conflict of superpowers based on nuclear power created political and intellectual possibilities for a non-military view of security. R. Stawicki [2] accurately described the direction of the concept of evolution as “towards man”.

The UNDP Report entitled, “The New Dimension of Human Security”, played an important role here [3]. It was the foundations of the concept of human security [4], i.e., security of a human being. The UN views human security in two significant aspects, as freedom from fear and freedom from wants, and lists seven specific categories: economic, food, environmental, personal, community, political and health. Thus, a new value appeared in activities for the health of societies—health security.

Despite the general recognition of the social importance of health security, there is no single common definition of this concept [5]. There are various and incompatible definitions of the concept and, thus, significant differences in understanding and using it in different settings [6]. That is of great inconvenience to communication regarding the subject, leading to the breakdown of cooperation and development, especially as a linkage between health security and health systems [7]. Hence, there is still a need for a generally accepted definition of health security that is neither simple nor easy. However, it becomes all the more difficult the longer there is no fundamental definition, i.e., a simple but comprehensive one, defining the issue’s essence, but leaving it open to possible further clarifications and the results of research.

The degree of interest in the health security problems of societies, scientific communities and public authorities is variable. It grows when the sense of security changes in different social circles. It often happens with increasing health problems, needs reformulation or deteriorating assessment of the functioning of health systems.

Such a process has been observed in recent years. The turning point was the Ebola epidemic, followed by problems with other infectious diseases, both new and re-emerging [8,9].

Until recently, it seemed that immunization and overall economic and social growth in most countries would lead to complete control of infectious diseases. However, this did not happen. In addition, the COVID-19 epidemic showed various deficiencies in the functioning of health systems affecting access to health care [10]. People were directly affected. Consequently, understanding the concept of health security and its determinants has become particularly important for recipients of health services.

Suppose we add that in Polish scientific literature on public health, the previous studies on health security appeared over 15 years ago [11,12]; in that case, it seems most justified to find an answer to the following questions: what is health security? How should this concept be understood at the beginning of the second decade of the 21st century, with particular emphasis on the subjective individual sense of security?

The study’s objective was to search, analyse and evaluate possible original propositions to define the concept of “health security” in Polish professional literature published from 10 January 2017 to 31 March 2022.

## 2. Methods

The research method was a scoping review [13] performed in five stages according to H. Arksey and L. O’Malley [14]. Scientific articles were searched for articles published between 1 January 2017 and 15 March 2022, in nine of the most important—according to the authors—Polish scientific journals in the field of public health or having broad reach in human resources for health community The journals are: *Przegląd Epidemiologiczny* (*Epidemiological Review*), *Hygeia Public Health*, *Zdrowie Publiczne i Zarządzanie* (*Health Public and Management*), *Zdrowie Publiczne* (*Public Health*), *Annals of Agriculture and Environmental Medicine*, *Problemy Higieny i Epidemiologii* (*Problems of Hygiene and Epidemiology*), *Lekarz Wojskowy* (*Military Physician*), *Gazeta Lekarska* (*Medical Journal*), *Nowiny Lekarskie* (*Medical News*). The nine most important Polish periodicals in military sciences and security are: *Bellona*, *Colloquium of the Faculty of Humanities and Social Sciences* of the Medical University of Warsaw, De Securitate et Defensione, *Przegląd Bezpieczeństwa Wewnętrznego* [*Internal Security Review*], *Przegląd Strategiczny* [*Strategic Review*], *Roczniki Bezpieczeństwa Międzynarodowego* [*Annals of International Security*], *Security and Defense Quarterly*, *Scientific Journal of Military University of Land Forces*, *Political Studies*.

The basis for the preliminary selection of studies was to find one of the following phrases in the title or the article: health security; human health security; patients’ security; patient security; human security; public health security.

The research question was: Has the original definition of health security appeared in the last five years? The definition was accepted as original when two prerequisites were checked: the author’s ascertainment in the text and non-repeating description from other sources.

## 3. Results

Twenty-eight studies were found that met the initial assumptions of health security. In the first approach, seven studies were rejected because they related to patient security in patient safety meaning [15,16,17,18,19,20,21]. Subsequently, another seventeen were denied, because the definition of health security was not actually discussed there or was limited to the literal quotation of other authors’ definitions [7,22,23,24,25,26,27,28,29,30,31,32,33,34,35,36]. Ultimately, the study criteria were met by four studies: P. Grzywna [37]; S. Jarmuszko [38]; J. Konieczny [39] and M. Paplicki [40].

In 2017, P. Grzywna’s monograph “Health security in social science. Introduction to the discussion” was published [39]. The author attempted to formulate the concept of health security, which—as he aptly noted—is one of the fundamental goals of the healthcare system, implemented primarily through the system of health care institutions, but also requiring both collective and individual activity.

In the first chapter, entitled, “Health security—preliminary findings”, the author considers the concept of “security” and proposes his own. The author suggests that health security should be defined as “ensuring conditions (social, economic and environmental) enabling the exercise of the right to health protection, an essential element of which is guaranteeing access to medical services on equal terms for beneficiaries by the state and its agencies. Therefore, access to the health care system is a factor that implies the level of health security”.

However, such a definition raises some doubts and practical questions. Suppose health security is the provision by the state and its agencies of conditions for implementing the right to health protection. In that case, the question is: who is to determine whether these conditions are met? If the state is to create such conditions, perhaps the state will also assess whether these conditions are met.

If P. Grzywna writes about ensuring health security by the state and its agencies, then he writes about ensuring it by public institutions. Therefore, are non-public institutions excluded from ensuring health security? After all, they have their place in the health system. It is worth quoting here the generally recognised definition of the health system contained in the Tallinn Charter. According to its wording, Within the political and institutional framework of each country, a health system is the ensemble of all public and private organisations, institutions and resources mandated to improve, maintain, or restore Health (WHO European Ministerial Conference on Health Systems “Health Systems, Health and Wealth”, Tallinn, Estonia, 25–27 June 2008: report.).

According to P. Grzywna, “health security means ensuring social, economic and environmental conditions (…)”. In pro-health activities, there are also organizational and political ones. There is no question that the sense of health security is influenced by the system of values the ruling power presents.

In the proposition under discussion, the conditions are specified by the phrase, “enabling the exercise of the right to health protection”. However, we would like to point out that the right to health protection is explicitly defined and guaranteed in Art. 68 of the Polish Constitution, which grants everyone the right to health protection. So, is the discussion on health security a debate about implementing the right to health protection? If so, then the whole discussion is transferred to a platform for deliberations on constitutional law and human rights.

An important element of health security—in the definition discussed—is for it to be a guarantee of access to medical services. Nevertheless, the health condition of societies is determined not only by access to health services but also—and perhaps above all—by the efficient functioning of public health services. Unfortunately, this issue was omitted. Additionally, the following question arise: what does an “essential” element of health security mean? Is it essential both qualitatively and quantitatively? At the same time, it should be remembered that a high number of health services does not automatically mean high quality. Moreover, if the availability of medical services implies a level of health security, then the question about their measures is justified. The greater the availability, the greater the security?

So, does total availability—and this is the favourite statement of politicians—mean total health security? Total access to health services is a myth, the same as full coverage of health needs. In health care, supply “drives” demand, and the “big equation”—that more significant expenditure on health care automatically improves the health of societies—has long been obsolete.

The point of reference for the health security definition proposed by P. Grzywna is the state and its agencies. However, health security should probably also be, and perhaps above all, talked about in the context of a sense of security, which is subjective and individual. This, in turn, raises questions about the health security of various economic and social entities.

In one of his latest studies, S. Jarmuszko writes about health security from the perspective of security anthropology [38].

It is supposed to be an approach that allows looking at health security from a different than usual point of view, i.e., from the perspective of security anthropology. However, the proposed description also raises several doubts. Health security—as we read—is the possibility for a person to maintain a “normal, proper and desirable state of physical and mental health in a favourable social environment”. If we read this literally, we can conclude that the very possibility of maintaining proper health is synonymous with health security. The question that needs to be resolved is: what is meant by proper health? Proper for whom? Individual or populational? Following that, should the “favourable social environment” be a condition sine qua non of health security? This is especially important since health care has never had an entirely conducive social environment.

According to this author, health and, thus, health security, “is determined by many external factors (ecology, social influences, politics or economy). And because, apart from the biomedical paradigm, there are other factors, with the sociological and socio-ecological ones at the forefront, the consequence is the extension of the essence of health security”.

It also covers “a number of environmental and even administrative factors, such as shaping pro-health attitudes, education and health promotion, prophylaxis, disease prevention, early diagnosis, effective treatment”. It also means “creating conditions conducive to health, strengthening pro-health social initiatives, developing individual skills to preserve and strengthen health, intensification of activities for health promotion and prophylaxis”. The concept of health security “also includes the broadly understood elimination of health risks, as well as palliative and hospice care”. Today, in the world of science and practice, after the significant publications of the World Health Organization, there is consensus as to the determinants of health, health risks, and elements of health systems [41,42]. These matters are exhaustively presented in the study by S. Jarmuszko. However, are we sure that their enumeration also determines health security?

In his study on public health security [39], J. Konieczny writes, “From a systemic perspective, public health security—of a population—is achieved when the state and its citizens are prepared to protect life and health in normal conditions and crisis situations through constant monitoring of threats, appropriate prophylaxis, taking the necessary actions to save human property and the environment, as well as ensuring the injured (sick) access to appropriate treatment centres (…) and restoring the environment to a state of equilibrium in accordance with the current state of medical knowledge based on evidence, legal basis and science about security”. Then, taking into account the concept of the “One Health” ecosystem, he believes that “other categories should be added to the definition of health security”. The only question is: what categories?

The quoted text is more like an enumeration, a description of tasks of the health system. The author assumes that health security is achieved when the state and its citizens are “prepared to protect health (…)”. What about the preparation period? So, is health security a state resulting from preparations? Additionally, what about the course of preparations for health protection? Additionally, who is to judge, and with what tools, that this condition has already been met? The state and the citizens are to be prepared. What if the state is prepared, and the citizens are not, or vice versa? Additionally, what about the apparent tendency to see the situation optimistically by the state and not by the citizens?

In his study on the health security of a citizen in the Polish emergency medical system, M. Paplicki [40] writes that “health security consists in refraining from refusing to provide health and life protection services in the event of a health emergency”. Additionally, a few sentences later, “health security is the certainty of the operation of government and local administration organisations and subordinate services in the life and health protection field. Moreover, health security is a process of continuous actions of states and individuals to satisfy their health needs”. Are these three different descriptions of the same concept? Moreover, all these descriptions have a carte blanche nature. Who is to refrain from refusing to provide benefits and on what legal basis? Probably, it is the so-called service provider. Suppose a citizen wants to define their health security level or think about it. In that case, it is hard to imagine that they would wonder who “abstained” from providing health services and whether the administration’s actions are characterised by certainty. If health security is a process of continuous efforts of the state and individuals to satisfy their health needs, there is the question of how to share the responsibility. How should it be measured?

## 4. Discussion

The study’s objective was to search, analyse and evaluate possible proprietary propositions to define the original definition of “health security” in Polish scientific literature on public health, military and security sciences. In the last five years, only in four studies did the original definition appear. E.g., the authors do not duplicate the generally known definitions of health security, trying to present a new perspective.

In the opinion of the authors of this study, it is difficult to find a breakthrough in the discussed studies regarding the definition of the term “health security”. Instead, they confirm the thesis that there is a need for a broad discussion on this subject, particularly regarding definitions.

One of the main reasons for the aforementioned “lack of breakthrough” is the failure to consider public health in the studies under discussion. Public health is a science with its perception of the problem and achievements in defining health, determinants, logic of actions for health, and an anthropocentric view of health security and its determinants. In this situation, the discussion about what “health security” is, is still justified. It seems, however, that there are issues the consideration of which would be preliminarily justified. For example, the issue of the general understanding of security [43] and the placement of health in security sciences. It is not easy to discuss health security without discussing security as such. Or maybe it is quite the opposite. Maybe adding the word “health” creates the concept’s utterly new quality and scope.

Today, security is considered in three categories: individual security (subjective dimension), security of social structures and international security [44]. This must be related to deontological considerations: is health security a real being, or is it an abstract product of the human mind?

It should be essential to decide whether the concept of health security refers only to human beings or also applies to institutions and organizations. The experience resulting from the operations for human health, both individual and populational, suggests the validity of the second approach. Health security concerns people and relates to the sphere of a sense of security. Sense is the inherent value of human life. Bestowing sense on an institution or organization evokes at least a desire for polemics. People who have created an entity that provides health security to individuals can experience a sense of security.

Health security is a sense of certainty for human beings to obtain medical help for themselves and their relatives when they know of such a need. Sense can be gradual, depending on the relationship between the conscious health need and the conscious expectation of its satisfaction. All other issues mentioned by the authors of the discussed studies as “components” of the definition of health security are determinants.

Adopting such an approach opens the field for research into what makes people have or not have a sense of security. It is an apparent didactic indication and a starting point for a discussion on the scope, methods and research tools regarding the sense of health security.

In this study, the situation in Poland was mainly discussed. However, it is a global problem. It should be for further investigation and comparison on the international level.

## 5. Conclusions and Propositions

1. No study on health security has appeared in Polish public health journals in the last five years.
2. The originally proposed definition of health security in chosen scientific sources failed to solve this problem and raised further questions and doubts.
3. It is urgent to start a discourse on the definition of the concept of “health security” with the broadest possible participation of representatives of various scientific disciplines, taking into account the knowledge and practice of public health. It would be worthwhile discussing the matter on the international level.
4. It seems that it will be impossible to avoid the following questions: what is health security nowadays? What is a personal issue? What are the necessary steps to achieve the consensus?
5. We recognise that health security is a sense, so it concerns only people. You will receive medical help for yourself and your loved ones if needed. Sense can be gradual, depending on the relationship between the conscious health need and the conscious expectation of its satisfaction.
6. All other matters used to define health security are nothing else than the determinants of health.

## Data Availability

All data are available from the corresponding author.
